# Fine-scale population spatialization data of China in 2018 based on real location-based big data

**DOI:** 10.1038/s41597-022-01740-5

**Published:** 2022-10-14

**Authors:** Mingxing Chen, Yue Xian, Yaohuan Huang, Xiaoping Zhang, Maogui Hu, Shasha Guo, Liangkan Chen, Longwu Liang

**Affiliations:** 1 Institute of Geographic Sciences and Natural Resources Research, CAS, Beijing, China; 2 College of Resource and Environment, University of Chinese Academy of Sciences, Beijing, China; 3 Institute of Urban and Rural Planning, China Academy of Building Research, Beijing, China

**Keywords:** Sustainability, Geography

## Abstract

Accurate location-based big data has a high resolution and a direct interaction with human activities, allowing for fine-scale population spatial data to be realized. We take the average of Tencent user location big data as a measure of ambient population. The county-level statistical population data in 2018 was used as the assigned input data. The log linear spatially weighted regression model was used to establish the relationship between location data and statistical data to allocate the latter to a 0.01° grid, and the ambient population data of mainland China was obtained. Extracting street-level (lower than county-level) statistics for accuracy testing, we found that POP2018 has the best fit with the actual permanent population (R^2^ = 0.91), and the error is the smallest (MSE_POP2018_ = 22.48 <MSE_WorldPop_ = 37.24 <MSE_LandScan_ = 100.91). This research supplemented in the refined spatial distribution data of people between census years, as well as presenting the application technique of big data in ambient population estimation and zoning mapping.

## Abstract (summary)

**Table:** 

Measurement(s)	population
Technology Type(s)	location-based big data
Factor Type(s)	spatial region
Sample Characteristic - Environment	spatial region
Sample Characteristic - Location	China

## Background & Summary

Human services and health^1,2^, disaster assessment^3,4^, global change^5^, infrastructure construction and urban planning^6^, human-environment coupling system^7^ and other applications rely heavily on population spatial data. The genuine population data originates from official census data, however there are several limitations in practical applications, such as difficult to achieve scale conversion, a long update time, and the inability to provide specifics about the population’s geographical distribution within administrative divisions^8^. It’s difficult to overlay census data with environmental data due to a lack of defined spatial references and consistent data units, which makes interdisciplinary study on human-environment systems limited^9^.

Early research used the population density model^10–13^ and different mathematical techniques of interpolation^14–18^ to mimic the population distribution inside a census data unit. The advancement of remote sensing and geographic information system (GIS) technology has opened up new possibilities for calculating spatial population distribution weights^19^. To obtain population data gridding and therefore increase accuracy and resolution^20–23^, several research included multi-source data and spatial variables such as land use and cover^24–26^, residential units^27,28^, transportation network^29^, night lights^30–32^. Many researchers are now combining GIS with computing technology to create intelligent models, such as random forest, genetic algorithms, multi-agent systems, and cellular automata^33–35^. This allows the model structure to be more flexible and the application scale to be more detailed.

Based on existing technological progress, widely-used data sets have been created internationally, such as the Gridded Population of the World (GPW)^36^, the Global Rural Urban Mapping Project (GRUMP)^37^, the Global Human Settlement Population Grid datasets (GHS-POP)^38^, the WorldPop^39^, and the LandScan^40^. Besides, the 1 km grid population dataset of China serves for China^41^. According to the data review, most of these datasets have long update periods, such as 5-year intervals^42^. Only a few datasets, including WorldPop and LandScan, provide continuous population data updated annually. And some years within the interval, such as 2018, lack widely available population datasets.

However, as a medium for refined population distribution, remote sensing-aided data are not a direct indication of population distribution and the intensity of human activity influence^34^, and refined population maps based on direct correlation of individual behaviors with refined global data are lacking. Second, current data are utilized to generate input population data, which is extrapolated from China’s 2010 county population census to target years using a county growth rate^38,43^. Every ten years, China conducts a population census. During this period, both the total population and the rate of growth change dramatically, therefore utilizing census data to forecast population in the middle years would result in substantial mistakes.

**Fig. 1:**
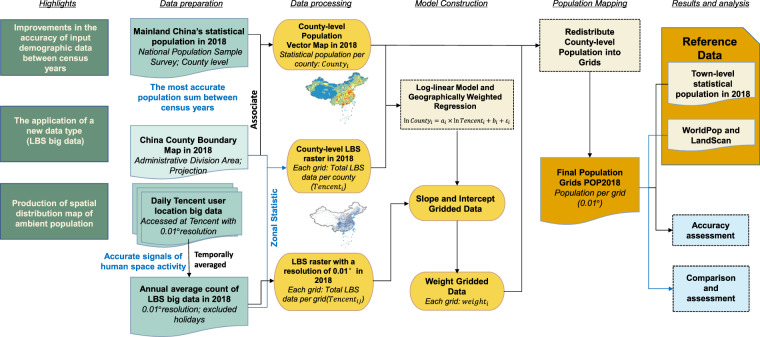
The research and production framework of population spatial distribution map.

To remedy these gaps, we present POP2018^44^, a gridded ambient population data set for mainland China in 2018 with 0.01° resolution. Large volumes of geospatial big data, such as mobile call data^45^ and traffic trajectory^46^, are utilized to estimate and simulate the geographical distribution of the population, attributable to the fast growth of mobile location-based services (LBS). Big data can help to improve social sensing and multiscale understanding of population distribution^47–49^. Some scholars have tried to use big data provided by Tencent, an internet company, as a social indicator in studies related to population distribution and mobility^50–52^. As illustrated in Fig. 1, we used the crawler to capture the real-time geo-location query number of user location given by Tencent’s location-based service (LBS) data and calculated the yearly average LBS data in 2018, which indicates that each grid population is a temporally averaged measure of population depending that POP2018 is the ambient population, according to Dobson *et al*.^23^. We utilized the National Bureau of Statistics of China’s 2018 Chinese mainland sample survey permanent population data, which is the most reliable demographic data in non-census years. The log linear spatially weighted regression model was used to establish the relationship between the two data, and the population number corresponding to the annual average LBS data in each grid was finally estimated.

## Methods

### The population data

Residential population statistics in mainland China were obtained from the National Bureau of Statistics 2018 national sample survey permanent population data with 2851 county-level units, equivalent to the level 3 of the global administrative unit layer, whose sample size accounts for about 1‰ of the country’s total population. The number of permanent residents, the name of the province, the city, the county, and the county’s administrative number are all included in the statistics. The permanent population refers to those who have lived in the county for more than six months and reflects the population’s real distribution. Population sample survey results are the most reliable permanent population data available in the non-census year. We also gathered data from the Dongguan Bureau of Statistics on town-level permanent populations. County-level permanent population data were utilized for regression model creation, while town-level data were employed for accuracy testing of population data products, as recommended by Gaughan *et al*.^53^.

### County administrative boundaries

**Fig. 2:**
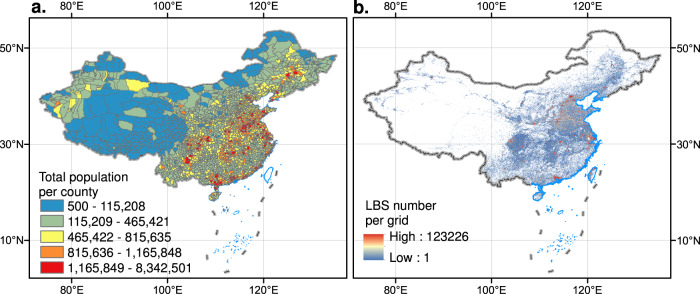
County-level permanent population (**a**) and Tencent positioning data (**b**).

The boundaries of China’s administrative divisions are downloaded from the national catalogue service for geographic information (www.webmap.cn). To create the 2018 county-based permanent population distribution map (Fig. 2a), assign sample survey population data to administrative divisions based on county names and administrative codes.

### User location big data

Location services provided by Tencent, an Internet company (https://cloud.tencent.com/solution/lbs) recorded the number of user location signals in grids with a spatial resolution of 0.01° and spatial reference GCS WGS84 every 5 minutes. Similar to Facebook and WhatsApp in the international market, Tencent is one of the most popular internet service provider in China, and its products (including WeChat, QQ, online maps, etc.) have over 1 billion users across 200 countries. More than 90% of the its’ users in 2018 are located in China^49^, covering people from all walks of life, different age groups and different regions. We use crawler technology to access Tencent’s positioning service in real time every 5 minutes, sum the positioning data for a day, and generate a spatial distribution map of daily positioning times, resulting in a total of more than 100 thousand maps of positioning data in 2018, including about 800 million online user’s data, with attributes such as time, longitude, latitude, and positioning times. We used the LZW-compression technique to save the map data in Geo-Tiff format for the analysis.

We used an arithmetic average to obtain average daily users location data from March to June and September to December in 2018, excluding the impact of Spring Festival transportation, students’ winter and summer vacations, holiday travel, when there is a large movement of people in China (Fig. 2b). Equation (1) is as follows:

1 $$Tencent=\frac{1}{n}{\sum }_{i=1}^{n}Count\_{d}_{i}$$

where *Tencent* is the average positioning count of Tencent big data in 2018, *Count_d*_*i*_ is the daily positioning counts of Tencent big data on day *i*, and *n* is the total number of non-holiday days from March to June and from September to December.

### Construction of a grid-scale population spatialization model

**Fig. 3:**
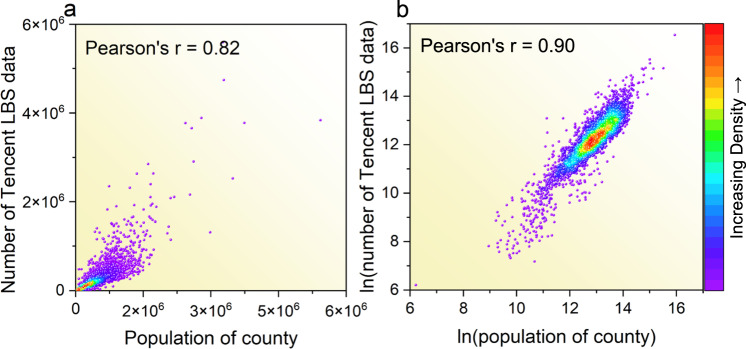
County-level statistical population and Tencent location number (**a**) and their logarithmic (**b**) kernel density plots.

The main statistical regression models we considered and compared include multiple linear regression^54^, polynomial regression^55^ and logarithmic linear model^56^, to fit the functional relationship between social perception data and census data^57,58^. The total number of Tencent user location big data in each county is calculated, which is then utilized for correlation analysis with the permanent population. The Pearson’s correlation coefficient between LBS big data and the permanent population is 0.82 (Fig. 3a). In the plot of linear fitting results (Fig. 3a), large scatters are concentrated in low values. After log-transformation of both the LBS count number and the permanent population, the correlation coefficient between them is 0.90 (Fig. 3b).

Considering the spatial correlation of population density, we constructed a logarithmic geographically weighted regression (GWR) model. The R^2^ of GWR is 0.91 (p < 0.05), which is higher than that of OLS (R^2^ = 0.81), and the residual sum of squares (RSS) of GWR (RSS = 201.78) lowers 224.5 when compared to OLS (RSS = 426.28). The local variable parameter model can better capture the geographic heterogeneous relationship between population distribution and Tencent positioning data. Therefore, a regionally weighted regression with local variable parameters can more accurately portray the pattern of smooth population change in local locations.

**Fig. 4:**
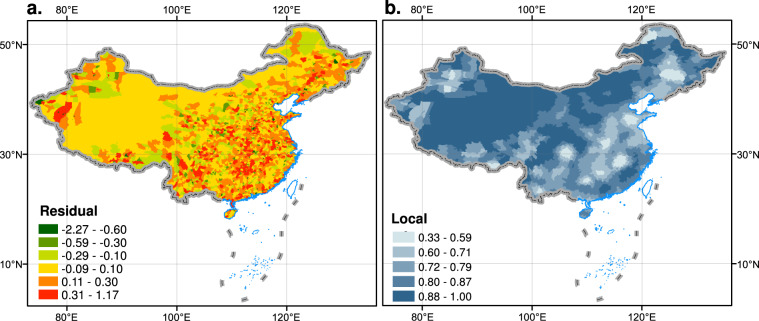
Geographically weighted regression fit residuals in counties (**a**) and local R² (**b**).

The log linear GWR model is used to fit demographic and Tencent data from 2851 county-level units in China, Eq. (2), which expresses the connection between the total number of Tencent positioning times at county-level and the permanent population at the end of the year:

2 $$ln\;Count{y}_{i}={a}_{i}\times ln\;Tencen{t}_{i}+{b}_{i}+{\varepsilon }_{i}.$$

where *Tencent*_*i*_ is the total number of daily positioning visits of Tencent big data in the *i*^th^ county-level region. *County*_*i*)_ is the permanent population at the end of the year 2018 in the *i*-th county. *a* is the superlinear impact of the number of residents at the end of the year on the total number of daily positioning visits of Tencent big data. *b* is scale ratio. *ε*_*i*_ is the residual and ${\varepsilon }_{i} \sim N(0,{\sigma }^{2}),\;Cov({\varepsilon }_{i},{\varepsilon }_{j})=0\;(i\ne j)$. We assume that the grid cells in each county have the same parameter. There are 1745 counties with the error between the estimated value and the actual value between −0.1 and 0.3, accounting for 61.2%, while only 12.3% of the counties in the central area have high residuals (residuals larger than 0.3 or less than −0.6) (Fig. 4a). The Local R^2^ is larger than 0.6 in 2674 counties, accounting for 93.8% of all counties, demonstrating that the GWR has an excellent local fitting impact.

### Population mapping

We use the built GWR model to estimate grid value by substitute the Tencent positioning data with a resolution of 0.01° in the Eq. (2). The demographics are redistributed by county using the estimates for each grid as weights, as shown in the Eq. (3):

3 $$pop201{8}_{ij}=\frac{Count{y}_{i}}{{\sum }_{j=1}^{n}weigh{t}_{ij}}\times weigh{t}_{ij}$$

where *pop2018*_*ij*_ represents the final population of the *j*^th^ grid in the *i*^th^ county. *weight*_*ij*_ represents the estimated value of the *j*^th^ grid in the *i*^th^ county from the GWR model. *County*_*i*_ is the population statistics of the *i*^th^ county. *n* is the total number of 0.01° grids in the *i*^th^ county, thus finally obtains the fine scale population spatial data POP2018.

### Accuracy assessment

We compared our result with the WorldPop^59^ and LandScan^60^ datasets, both of which have a resolution of 30 arc. The unconstrained gridded population data of WorldPop is used in this research. LandScan utilizes sub-national census counts given by the International Program Center, Bureau of Census, whereas WorldPop uses county totals based on China’s 2010 county population census data. Both WorldPop and POP2018 adopt the “top-down” population spatialization idea. Comparing with the widely recognized data from WorldPop can help us understand the difference of spatial distribution depicted by ambient and residential population. We collected the permanent population of 33 towns provided by the Dongguan Bureau of Statistics, which is the population who lived in each town for more than half a year. We use the town-level population as validation data to test the accuracy of POP2018. According to Ye^34^ *et al*. (2019), we compared mean square error and goodness of fit. We also selected cities with a population of less than 5 million (Huangshi), 5 to 10 million (Xi’an), and more than 10 million (Shanghai), and compared the details of the population distribution of the three data in these three cities.

## Data Records

Table 1 shows the data involved in the article. The 0.01° grid population data of China mainland in 2018 can be accessed freely at the figshare repository^44^ (10.6084/m9.figshare.20400717.v1). The data collection contains one.rar file, labelled China_POP_0.01deg_2018.rar. It contains two GEOTIFFs and a package of a polygon feature, which are the annually average Tencent LBS data in 2018, the 0.01° grid population data of China mainland in 2018 and the county-level boundary map joining with statistical population in 2018. All data were mapped using the Albers equal-area projection. The original LBS data files are saved as text JSON file. Due to the fine temporal resolution (5 minutes), the amount of the original dataset too huge to upload, which can be requested from corresponding authors.

**Table 1:** Categories of data used to fit the model and evaluate the accuracy of the new population density map.

Dataset	Format	Source	Reference link
National Population Sample Survey data (2018)	Excel	National Bureau of Statistics	http://www.stats.gov.cn/tjsj/ndsj/2019/indexeh.htm
Tencent user location big data	GEOTIFF	Tencent Cloud	The real-time data application can be accessed at https://cloud.tencent.com/solution/lbs, and the annually average user location data in 2018 compiled in this article can be obtained from 10.6084/m9.figshare.20400717.v1^44^
Boundary maps	Polygon features	National Catalogue Service for Geographic Information	www.webmap.cn
LandScan (2018)	Raster	Oak Ridge National Laboratory	https://landScan.ornl.gov/
WorldPop China Mainland (2018)	Raster	WorldPop, School of Geography and Environmental Science, University of Southampton	10.5258/SOTON/WP00674^62^

**Fig. 5:**
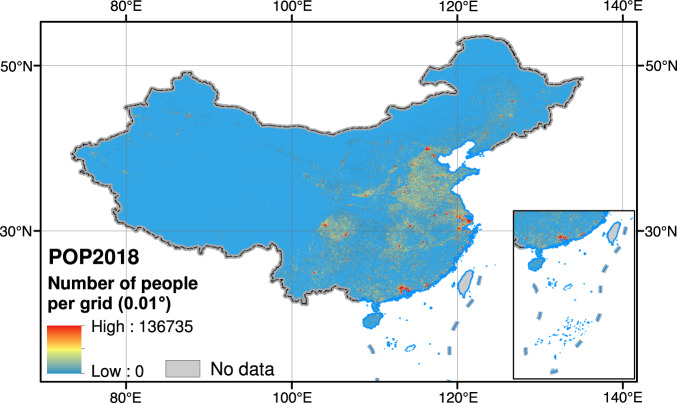
0.01° resolution spatial population data for 2018 across mainland China (POP2018 dataset).

The county-level population sample survey and the Tencent position big-data are used to create a high-resolution gridded population distribution dataset for China (2018). The grid value in Fig. 5 reflects the individuals who have been physically distributed in the grid for more than half a year, and the unit is person. The dataset with spatial reference GCS WGS84 given in GeoTiff format, closely portrays the geographical distribution pattern of people in China (2018). It demonstrates that the population distribution presents a clustered distribution pattern, forming multiple population hotspots (red dots). Larger hotspots are located in urban agglomerations with a high level of modernization and urbanization, such as the Yangtze River Delta and the Pearl River Delta, as well as large cities such as Beijing, Tianjin, Chengdu and Chongqing. The area of the hotspot can represent the population scale level, showing the hierarchical distribution of the population among towns. The North China Plain, the Sichuan Basin and the middle and lower reaches of the Yangtze River all have relatively dense small and medium-sized hot spots, showing a relatively dense urban spatial system in the plain area. In the suburbs or between cities, the population distribution is mainly distributed along the traffic lines and presents a network shape, which also reflects the actual situation that human activities on the traffic network are stronger than farmland in the outer suburbs. POP2018 not only reflects the dispersed population distribution caused by mountainous and hilly areas in southeastern China, but it also outlines the main population distribution areas in northwestern China, which are squeezed by a large area of plateau, deserts, and large mountains, such as the Gansu Corridor, the southern piedmont of Tianshan Mountain, and the oasis area around the Taklimakan Desert and the southern Tibet Valley.

**Fig. 6:**
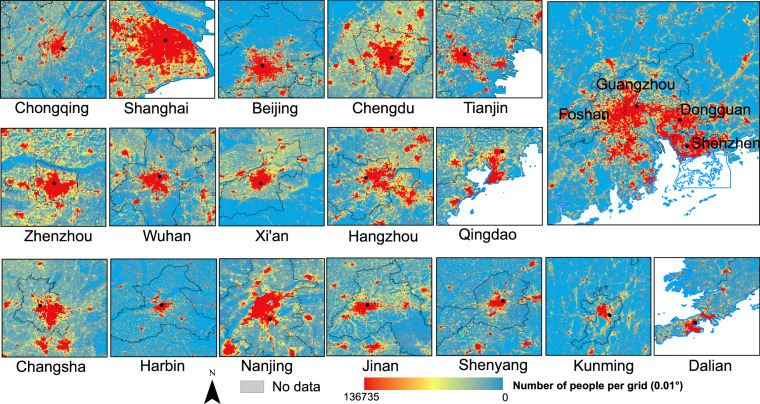
Estimated population spatial distribution in cities with population of more than 5 million.

We select cities with a population size greater than 5 million to zoom in, more details can be observed from Fig. 6. The population is mostly concentrated in the city’s center region, indicating a development pattern of extending from the core to the periphery. For large cities in the central and western China with a large agricultural population, such as Chengdu, Chongqing, and Changsha, high-density central urban areas, there are more areas in the transition stage of population on the fringes. While some large cities in the eastern coastal areas, such as Beijing, Shanghai, etc., have a high-density population core area in stark contrast with the sparsely populated suburbs, which might bring problems such as congestion in the central city and high housing pressure. Cities in the northeastern China, such as Harbin, Shenyang and Dalian, the population is concentrated in the city center, and the connections with surrounding towns are less apparent. The population distribution map provides a more accurate basis for understanding the current situation of urban development and urban system planning.

## Technical Validation

**Fig. 7:**
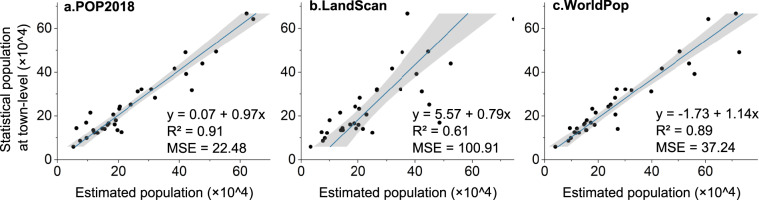
Scatter plot of POP2018 (**a**), LandScan (**b**) and WorldPop (**c**) and Dongguan township statistical population.

POP2018 has the smallest error and the highest accuracy between the population allocated to the town and the statistical permanent population data (Fig. 7). The population estimation errors of landscan and worldpop are smaller in sparsely populated towns, while the errors increase as the population increases (Fig. 7b,c). It can be seen that the distribution of POP2018 to the urban center area with agglomeration shows the advantages of positioning big data as auxiliary data. The coefficient between the population allocated to each town by POP2018 and the actual permanent population of each town reaches 0.97, which is approximately equal, while WorldPop and LandScan have slopes of 1.14 and 0.79. Based on the slope, WorldPop underestimated the population of most towns, which would cause it to over-allocate the population to one or two towns located in the center of the city. Conversely, LandScan overestimated the population of most towns. Both POP2018 and WorldPop fit the actual permanent population well (${{\rm{R}}}_{POP2018}^{2}=0.91$, ${{\rm{R}}}_{WorldPop}^{2}=0.89$). The mean square error (MSE) of POP2018 is the smallest at 22.48, indicating the smallest deviation between the estimated value and the actual value. In the towns with larger population, the advantages of POP2018 are shown, and the errors are smaller than those of the other two data. The estimation errors for both POP2018 and worldpop for towns with a statistical population of less than 400,000 are small.

**Fig. 8:**
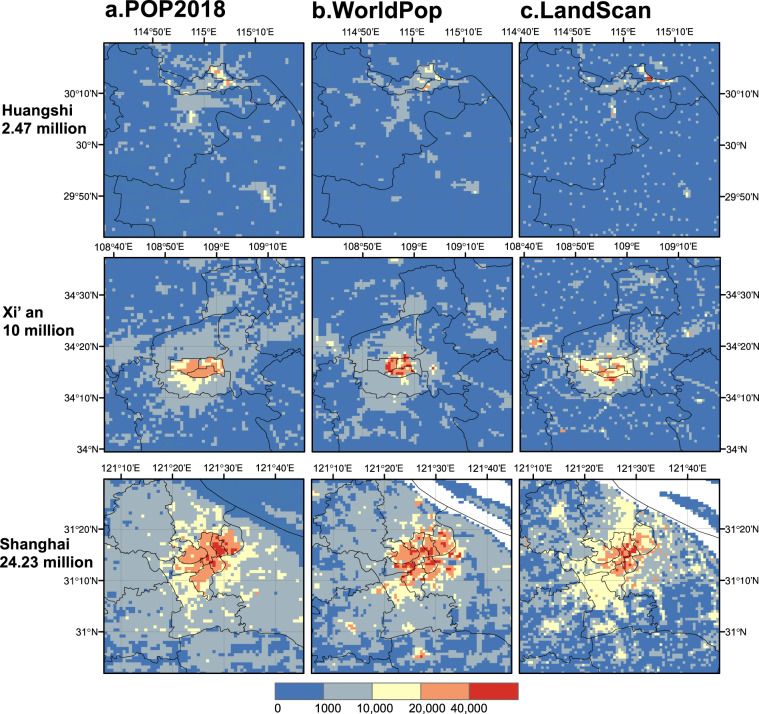
Population distribution of POP2018 (**a**), WorldPop (**b**) and LandScan (**c**) in the three cities of Huangshi, Xi’an and Shanghai.

We compare the detailed characterization of cities with different population sizes in the three datasets (Fig. 8). The central locations of the clusters of high-population areas estimated by the three data are roughly similar, and different data are sensitive and consistent in identifying urban areas with high population density. These orange and red areas are smaller in size than the blue areas, but have an order of magnitude higher population, reflecting the large difference in population densities between urban and rural areas. The difference between the three data on the distribution of urban population is that WorldPop assigns the highest population to the central urban area. The number of red grids in Xi’an and Shanghai is significantly more than other data. In comparison, LandScan underestimates the urban population due to the fact that the number of grids with a value between 20,000 and 40,000 is considerably less than other data. POP2018 balances the performance of WorldPop and LandScan in the city center, assigning a moderate number of grids with unusually high population.

For sparsely populated outer suburbs and rural areas, POP2018 and LandScan identify population settlements closer, while in terms of population buffers with population values in the middle, the distribution characteristics of POP2018 and WorldPop are similar. In rural areas, both POP2018 and LandScan grids with a population of 1,000 to 10,000 show scattered settlements (Fig. 8a,c), while WorldPop’s discrete spatial pattern is not obvious in comparison. Areas with a population of 1,000 to 10,000 around the central area of the city can be regarded as the suburban areas of the city, which are the transition areas from towns to villages. Both POP2018 and WorldPop show a circle structure that spreads outward, and the boundaries are similar (Fig. 8a,b). POP2018 combines the characteristics of WorldPop and LandScan in the display of population space, which not only has the characteristics of scattered distribution of rural settlements, but also is consistent with WorldPop’s high-density population distribution boundary in cities.

## Usage Notes

This paper provides a population data and production method of ambient population, which is defined as the time average of population, taking into account activities such as human work, shopping, eating, and traveling^61^, which can better reflect characteristics of population distribution than residential-based population data. For example, central business districts have a higher concentration of human activity than residential neighborhoods, despite the former being less inhabited. The production and application of environmental population is the future development direction of population spatiotemporal distribution research.

The POP2018 can be applied to overlaying analysis with natural environment data such as land use, vegetation index, night light, and DEM, facilitating the study of interdisciplinary fields of nature and the humanities. Simultaneously, the problem of collinearity between the provided population and other spatial data can be also effectively avoided in this application, given that the weight of POP2018 is calculated based on Tencent’s user location data independent of other environmental data.

WorldPop and LandScan have high influence and reference value, who have produced global population grid data to fill the vacancy of population spatial information of countries or regions with missing statistical data. The comparison with WorldPop and LandScan shows that the data and methods provided in this paper are more accurate and precise in estimating the population distribution in China, especially at the scale below the county level, illustrating the advantages of local scholars and institutions in spatializing the local population distribution. These local scholars and research have a better understanding of their respective national conditions and can obtain more suitable methods and reliable input data, which effectively guarantees the quality of data products from the source and shorten production cycle of making a global population distribution map. We believe it makes sense to establish a data providing platform composed of local data produced by local research institutions.

The provided data fills in the gaps in fine-scale population distribution data between census years. Using sample survey statistics has a smaller error than the population calculation based on the growth rate, and is more in line with the actual situation in China, which makes it possible to update population data annually. Since there are spatial differences in the fitting effect of the GWR model and our data validation has not been completed nationwide, it is recommended that users estimate the variation in accuracy in different geographies when using the data. In population spatialization technology, the merging of demographic data and big data is being investigated. Unlike the usually employed indirect cofactors, big data is created directly by people, which more precisely represents the actual situation of population distribution and opens up new possibilities for fine-scale population spatialization^48^.

## Data Availability

The programs used to generate the data were GWR4 and ArcGIS (10.7). The code is available on GitHub (https://github.com/Yue121/0.01deg-population-China-2018).

## References

[1] Hay SI, Noor AM, Nelson A, Tatem AJ (2005). The accuracy of human population maps for public health application. Trop Med Int Health.

[2] Dong N, Yang X, Cai H, Xu F (2017). Research on grid size suitability of gridded population distribution in urban area: A case study in urban area of Xuanzhou district, China. PloS one.

[3] Aubrecht C, Özceylan D, Steinnocher K, Freire S (2013). Multi-level geospatial modeling of human exposure patterns and vulnerability indicators. Natural Hazards.

[4] Ahola T, Virrantaus K, Krisp JM, Hunter GJ (2007). A spatio‐temporal population model to support risk assessment and damage analysis for decision‐making. International Journal of Geographical Information Science.

[5] Blankespoor B, Dasgupta S, Lange G-M (2017). Mangroves as a protection from storm surges in a changing climate. Ambio.

[6] Jia P, Qiu Y, Gaughan AE (2014). A fine-scale spatial population distribution on the High-resolution Gridded Population Surface and application in Alachua County, Florida. Applied Geography.

[7] Sutton P, Roberts D, Elvidge C, Baugh K (2001). Census from Heaven: An estimate of the global human population using night-time satellite imagery. Int. J. Remote Sens..

[8] Mao H, Ahn Y-Y, Bhaduri B, Thakur G (2017). Improving land use inference by factorizing mobile phone call activity matrix. Journal of Land Use Science.

[9] Zandbergen PA, Ignizio DA (2010). Comparison of Dasymetric Mapping Techniques for Small-Area Population Estimates. Cartography and Geographic Information Science.

[10] Sutton P, Roberts C, Elvidge C, Meij H (1997). A comparison of nighttime satellite imagery and population density for the continental united states. Photogramm. Eng. Remote Sens..

[11] Batty, M. & Longley, M. *Fractal Cities - A Geometry of Form and Function*. (1994).

[12] Newling BE (1969). The spatial variation of urban population densities. Geogr. Rev..

[13] Clark C (1951). Urban population densities. J. R. Stat. Soc. Ser. A-Stat. Soc..

[14] Reibel M, Agrawal A (2007). Areal interpolation of population counts using pre-classified land cover data. Popul. Res. Policy Rev..

[15] Mennis J (2003). Generating surface models of population using dasymetric mapping. Prof. Geogr..

[16] Fisher PF, Langford M (1996). Modeling sensitivity to accuracy in classified imagery: A study of areal interpolation by dasymetric mapping. Prof. Geogr..

[17] Holt JB, Lo CP, Hodler TW (2004). Dasymetric estimation of population density and areal interpolation of census data. Cartogr. Geogr. Inf. Sci..

[18] Yuan Y, Smith RM, Limp WF (1997). Remodeling census population with spatial information from LandSat TM imagery. Comput. Environ. Urban Syst..

[19] Tan M, Liu K, Liu L, Zhu Y, Wang D (2017). Spatialization of population in the Pearl River Delta in 30 m grids using random forest model. Progress in Geography.

[20] Li X, Zhou W (2018). Dasymetric mapping of urban population in China based on radiance corrected DMSP-OLS nighttime light and land cover data. Sci. Total Environ..

[21] Wang L (2018). Mapping population density in China between 1990 and 2010 using remote sensing. Remote Sens. Environ..

[22] Yang X, Huang Y, Dong P, Jiang D, Liu H (2009). An updating system for the gridded population database of China based on remote sensing, GIS and spatial database technologies. Sensors.

[23] Dobson JE, Bright EA, Coleman PR, Durfee RC, Worley BA (2000). LandScan: a global population database for estimating populations at risk. Photogramm. Eng. Remote Sens..

[24] Li S, Juhasz-Horvath L, Harrison PA, Pinter L, Rounsevell MDA (2016). Population and age structure in Hungary: a residential preference and age dependency approach to disaggregate census data. J. Maps.

[25] Dmowska A, Stepinski TF (2017). A high resolution population grid for the conterminous United States: The 2010 edition. Comput. Environ. Urban Syst..

[26] Batista e Silva F (2020). Uncovering temporal changes in Europe’s population density patterns using a data fusion approach. Nat. Commun..

[27] Dong P, Ramesh S, Nepali A (2010). Evaluation of small-area population estimation using LiDAR, Landsat TM and parcel data. Int. J. Remote Sens..

[28] Lu Z, Im J, Quackenbush L, Halligan K (2010). Population estimation based on multi-sensor data fusion. Int. J. Remote Sens..

[29] Zhao M, Liu S, Qi W (2017). Exploring the differential impacts of urban transit system on the spatial distribution of local and floating population in Beijing. J. Geogr. Sci..

[30] Amaral S, Monteiro AM, Câmara G, Quintanilha J (2006). DMSP/OLS night‐time light imagery for urban population estimates in the Brazilian Amazon. International Journal of Remote Sensing.

[31] Elvidge CD, Baugh KE, Anderson SJ, Sutton PC, Ghosh T (2012). The Night Light Development Index (NLDI): a spatially explicit measure of human development from satellite data. Social Geography.

[32] Zhuo L (2009). Modelling the population density of China at the pixel level based on DMSP/OLS non‐radiance‐calibrated night‐time light images. International Journal of Remote Sensing.

[33] Xiao H, Tian H, Zhu P, Yu H (2010). The dynamic simulation and forecast of urban population distribution based on the multi-agent system. Progress in Geography.

[34] Ye T (2019). Improved population mapping for China using remotely sensed and points-of-interest data within a random forests model. Science of The Total Environment.

[35] Cheng, Z., Wang, J. & Ge, Y. Mapping monthly population distribution and variation at 1-km resolution across China. *International Journal of Geographical Information Science* (2020).

[36] Tobler W, Deichmann U, Gottsegen J, Maloy K (1997). World population in a grid of spherical quadrilaterals. International journal of population geography: IJPG.

[37] Balk, D. L. *et al*. in *Adv. Parasitol*. Vol. 62 (eds S. I. Hay, A. Graham, & D. J. Rogers) 119–156 (Academic Press, 2006).

[38] Freire, S., Macmanus, K., Pesaresi, M., Doxsey-Whitfield, E. & Mills, J. Development of new open and free multi-temporal global population grids at 250 m resolution. *Population* **250** (2016).

[39] Tatem AJ (2013). Quantifying the effects of using detailed spatial demographic data on health metrics: a systematic analysis for the AfriPop, AsiaPop, and AmeriPop projects. The Lancet.

[40] Bhaduri B, Bright E, Coleman P, Urban ML (2007). LandScan USA: a high-resolution geospatial and temporal modeling approach for population distribution and dynamics. GeoJournal.

[41] Fu, J., Jiang, D. & Huang, Y. 1 km grid population dataset of China (2005, 2010). *Glob. Chang. Res. Data Publ. Repos* (2014).

[42] Leyk S (2019). The spatial allocation of population: a review of large-scale gridded population data products and their fitness for use. Earth System Science Data.

[43] Doxsey-Whitfield E (2015). Taking advantage of the improved availability of census data: a first look at the gridded population of the world, version 4. Papers in Applied Geography.

[44] Chen M (2022). figshare.

[45] Kang C, Liu Y, Ma X, Wu L (2012). Towards estimating urban population distributions from mobile call data. Journal of Urban Technology.

[46] Cai H, Jia X, Chiu AS, Hu X, Xu M (2014). Siting public electric vehicle charging stations in Beijing using big-data informed travel patterns of the taxi fleet. Transportation Research Part D: Transport and Environment.

[47] Liao C (2018). Big data-enabled social sensing in spatial analysis: Potentials and pitfalls. Trans. GIS.

[48] Yao Y (2017). Mapping fine-scale population distributions at the building level by integrating multisource geospatial big data. Int. J. Geogr. Inf. Sci..

[49] Hu M (2019). Visualizing the largest annual human migration during the Spring Festival travel season in China. Environment and Planning A: Economy and Space.

[50] Xu Y, Song Y, Cai J, Zhu H (2021). Population mapping in China with Tencent social user and remote sensing data. Applied Geography.

[51] Pan J, Lai J (2019). Spatial pattern of population mobility among cities in China: Case study of the National Day plus Mid-Autumn Festival based on Tencent migration data. Cities.

[52] Song J, Tong X, Wang L, Zhao C, Prishchepov AV (2019). Monitoring finer-scale population density in urban functional zones: A remote sensing data fusion approach. Landscape and urban planning.

[53] Gaughan AE (2016). Spatiotemporal patterns of population in mainland China, 1990 to 2010. Scientific Data.

[54] Chun J, Zhang X, Huang J, Zhang P (2018). A Gridding Method of Redistributing Population Based on POIs. Geography and Geo-information Science.

[55] Wu Z, Xu H, Hu Z (2019). Fine-Scale Population Spatialization Based on Tencent Location Big Data: A Case Study of Moling Subdistrict,Jiangning District,Nanjing. Geography and Geo-information Science.

[56] Zhang L, Jiale Q, du Y, Yi J, Sun Y (2020). Multi-level Spatial Distribution Estimation Model of the Inter-regional Migrant Population Using Multi-source Spatio-temporal Big Data: A Case Study of Migrants from Wuhan during the Spread of COVID-19. International Journal of Geo-Information.

[57] Deville P (2014). Dynamic population mapping using mobile phone data. PNAS.

[58] Khodabandelou G, Gauthier V, Fiore M, El-Yacoubi MA (2018). Estimation of static and dynamic urban populations with mobile network metadata. IEEE Transactions on Mobile Computing.

[59] WorldPop (School of Geography and Environmental Science, U. o. S. D. o. G. a. G., University of Louisville; Departement de Geographie, Universite de Namur) and Center for International Earth Science Information Network (CIESIN), Columbia University Global High Resolution Population Denominators Project - Funded by The Bill and Melinda Gates Foundation (OPP1134076). (2018).

[60] Rose, A. N., McKee, J. J., Urban, M. L., Bright, E. A. & Sims, K. M. LandScan 2018. (2019).

[61] Sutton PC, Elvidge C, Obremski T (2003). Building and evaluating models to estimate ambient population density. Photogrammetric Engineering and Remote Sensing.

[62] WorldPop, Bondarenko M (2020). University of Southampton.

